# Substance use disorders in Saudi Arabia: a scoping review

**DOI:** 10.1186/s13011-020-00285-3

**Published:** 2020-06-17

**Authors:** Nazmus Saquib, Ahmad Mamoun Rajab, Juliann Saquib, AbdulRahman AlMazrou

**Affiliations:** College of Medicine, Sulaiman Al Rajhi University, PO Box 777, Bukairyah, Al-Qassim 51941 Saudi Arabia

**Keywords:** Substance use disorder, Saudi Arabia, Quality, Review

## Abstract

**Background:**

Substance use disorders (SUD) are mental health conditions that arise from chronic drug use. There is an increased recognition of this problem in Saudi Arabia.

**Objective:**

Conduct a comprehensive review of published literature on SUD to identify knowledge gaps and to guide future research.

**Methods:**

PubMed, Embase and Cochrane databases were searched with suitable keywords for SUD publications up to June 10, 2019. Eligible studies (primary research conducted in Saudi Arabia) were organized into three broad domains: (1) risk (or protective) factors of SUD, (2) perspectives on drug use of people who use drugs, and (3) impact on family. The quality of the included studies was assessed with the Newcastle-Ottawa Scale.

**Results:**

Of the 113 search records, 23 were eligible for analysis (19 cross-sectional and 4 case-control). All studies were conducted in clinical settings; all but two included males only. There were 4 studies about SUD risk factors, 6 studies about the perspectives of people who use drugs, and none about family impact. None of the cross-sectional studies (0%) and 25% of case-control studies were of good quality.

**Conclusions:**

The available studies were few in number, weak in methodology, and poor in quality. Quantitative as well as qualitative studies about SUD are warranted in each domain and should represent both genders.

## Introduction

Substance (drug) use disorder (SUD) is a medical condition that arises from chronic drug use and is characterized by a cluster of cognitive, behavioral, and physiological symptoms that a person who uses drugs (PWUD) exhibits [1]. A hallmark of this condition is that the PWUD continues to use the drug despite experiencing significant negative consequences. SUD renders huge physical, psychological, and financial costs to the PWUD, the family members, and the society at large [2]. According to the latest United Nations 2019 Drug Report (UNODC), an estimated 271 million people (5.5% of the global population, aged 15–64) had used drugs in the previous year [3]. Additionally, 35 million people were diagnosed with and received treatment services for SUD [3].

Saudi Arabia is an Islamic country, and the societal norms and values are deeply rooted in religion. There are religious as well as legal prohibitions against possession or consumption of alcohol and narcotic substances, yet a portion of Saudis consume alcohol and use drugs [4]. Around 7 to 8% of Saudis report having used drugs [5, 6]; 70% of all PWUDs are 12–22 years old [5]. The most commonly abused substances among Saudis are amphetamines, heroin, alcohol, and cannabis, and a majority of PWUDs are addicted to multiple substances [7]. Over the past decade, the use of cannabis and amphetamines has increased, while the use of heroin and volatile substances has decreased [4, 8]. A portion of Saudi females also uses drugs, and the usage among them may be on the rise [9]. Drugs are not as accessible to women as they are to men in Saudi Arabia due to the conservative nature of the society and the strict gender segregation. Therefore, women are prone to using primitive and volatile substances such as glue, gasoline, and shisha [9].

The existing data on SUD among Saudis are outdated and non-specific. According to the World Health Organization, 0.01% of men ≥15 years of age had substance use disorders (2004 estimate) [10, 11]. Nearly 10,000 Saudis inject drugs; prevalence of human immunodeficiency virus (HIV), hepatitis C (HCV), and hepatitis B (HBV) among them were 3.5, 77.8 and 7.7%, respectively [12]. The SUD estimate is likely an underestimation because of the stigma and fear of disclosure associated with substance use. There is indirect evidence that points to a growing SUD problem. For example, more centers have been established in major cities, and a large budget has been allocated (one billion dollars per year) for the treatment and rehabilitation of PWUDs [13].

Hence, a multidimensional understanding of SUD is needed at this point in order to develop a comprehensive program to manage this problem in the society. Some of the major dimensions include risk (or protective) factors for SUD, perspectives of PWUDs, treatment and rehabilitation, and the impact on family members. International studies provide evidence for each of these dimensions. For example, family and religious values play an important protective role in drug use [14–16], common motivations for drug use include curiosity, relaxation, and peer pressure [17], knowledge about drugs and their effects depend on users’ age and education [18], and substance use takes a physical (e.g., violence), psychological (e.g., depression, break-up of relationship), and financial toll on family members [19, 20].

Therefore, a comprehensive review on SUD research in Saudi Arabia is timely and can be the starting point for understanding this problem for both the policymakers and local researchers. The most recent review on substance use in Saudi Arabia was by Bassiony in 2013 [4]. It was not specific to SUD but rather described substance use in general over the preceding two decades. It also did not assess the methodological quality of the included studies. The rationale for the current review is that the absolute number of people who have a substance use problem is likely high in Saudi Arabia because its demographic distribution is heavily tilted toward youth (around 15% of the total population is between 15 and 24 years old) [21], and youth are most affected by substance use. This review is also important in the context of the changing nature of Saudi society, which has traditionally been deeply religiously conservative, sustained by the Islamic principles of balance, restraint, and modesty. Its values are challenged in this information age, where countries are connected in the virtual realm to western cultures that promote individual identity, glorify drug use, and peddle pop culture. Saudi youth is most affected by this clash of cultures. They are increasingly alienated from their relatively more traditional parents, and it is in this vacuum where societal problems like substance use likely arise.

This review compiled all SUD-related publications from Saudi Arabia until the present. The specific objectives were to (1) describe the characteristics of the published studies, (2) assess their methodological quality, (3) identify areas where there is limited research evidence, and (4) make specific recommendations for future SUD research. The findings of this paper will help policymakers identify priority areas for SUD research and will guide prospective researchers in designing studies in areas that are currently deficient.

## Methods

### Literature review and data sources

Three databases were searched in June of 2019: PubMed, Embase and Cochrane Controlled Register of Trials (CENTRAL). Studies published up until June 10, 2019 were included. The search terms used in PubMed were (“Substance-Related Disorders”[Mesh]) AND “Saudi Arabia”[Mesh]). The Medical Subject Heading (MeSH) was used in both PubMed and CENTRAL and the search terms were (“Substance-Related Disorders”[Mesh]) AND “Saudi Arabia”[Mesh]). A multi-field search was used in Embase to search the following: “substance use disorder” AND “Saudi Arabia”. Additionally, national journals (Saudi Medical Journal and Annals of Saudi Medicine) and the reference lists of eligible articles were searched to identify additional published studies.

### Eligibility criteria

All published studies that (1) either included patients with SUD as the sample or used SUD as an outcome, and (2) were conducted in Saudi were eligible for review (i.e., inclusion criteria). Publications about SUD that did not stem from primary research (e.g., opinion, letters to editor) as well as conference proceedings or abstracts were not included (i.e., exclusion criteria).

### Search outcomes

**Fig. 1:**
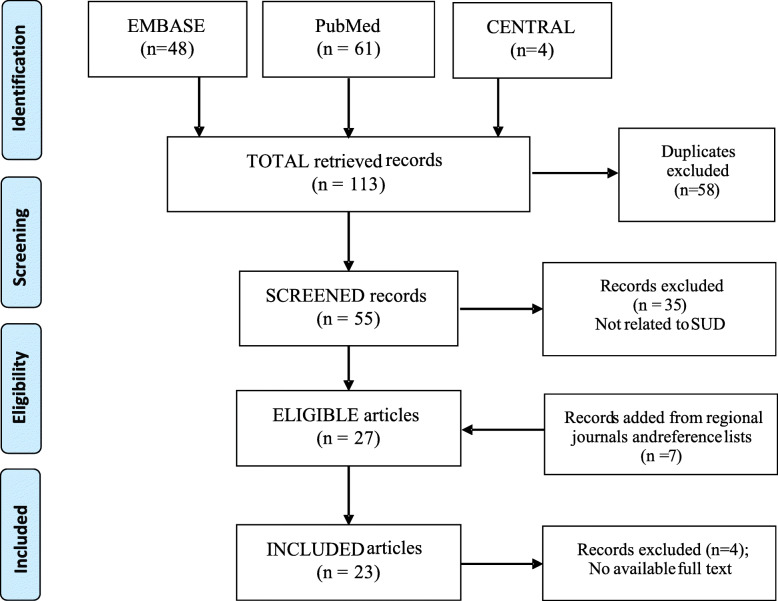
Flowchart of the eligible trials

Respectively, 61, 48, and 4 records were retrieved from PubMed, Embase, and CENTRAL (total = 113). These produced 55 unique records after the duplicates (*n* = 58) were removed through a careful read of the study titles. They were screened, and 35 did not meet the inclusion criteria, producing 20 eligible articles. An additional 7 articles that met the eligibility criteria were found after searching the national journals and scanning the reference lists of the primary 20 articles (total eligible =27). The full texts of 4 articles could not be retrieved despite making efforts (e.g., contacting the authors via e-mail) [22–25]. The total articles included in this review were therefore, 23 (Fig. 1).

### Data extraction process

From each included study, data were abstracted on (1) authors’ names, (2) publication year, (3) study design, (4) sample age and size, (5) study population, (6) location, (7) criteria used to diagnose SUD, and (8) main findings. Initially, the data of the included studies were charted by one co-author and then were reviewed independently by the lead and senior authors. Any discrepancy was resolved through discussion and consensus among the authors.

### Quality assessment of the included studies

The quality of the eligible studies was evaluated using the Newcastle-Ottawa Scale (NOS) for both cross-sectional (adapted from the cohort scale) and case-control studies [26, 27] (*see**supplementary materials**)*. The scale (7 items for cross-sectional, and 8 items for case-control) uses a star system for evaluation of studies in three broad areas: the selection of the study groups, the comparability of the groups, and the ascertainment of either the outcome or exposure of interest for cross-sectional and case-control, respectively. Cross-sectional studies were classified from the summary score as very good (9–10), good (7–8), satisfactory (5–6) and unsatisfactory (0–4) [26]. The cut-off values for case-control studies were slightly different: very good (7–9), good (5–6), satisfactory (4) and unsatisfactory (0–3) [27].

### Data analysis

Characteristics of the included studies, along with the main results, were tabulated and categorized under broad themes. In addition, cross-sectional and case-control studies were summarized separately according to NOS quality indicators. Finally, the frequency distribution of studies by quality was calculated and charted.

## Results

### General description of the studies

Of the 23 eligible studies, 19 (83%) were cross-sectional and 4 (17%) were case-control [7, 9, 17, 28–47]. The first study on SUD was published in 1992, and the successive three decades since then (i.e., 1992–2000, 2001–2010 and 2011–2019) saw approximately equal proportions of publications (33%). The majority of these studies were conducted in the western region (*n* = 11, 48%) followed by the eastern (*n* = 7, 30%) and the central region (*n* = 5, 22%). Only 2 studies included females [9, 17]. All studies obtained their samples from hospitals (e.g., Al-Amal Mental Hospital Complex). The sample size ranged between 101 and 12,743. Fewer than half of the studies (48%) used an established diagnostic tool (e.g., DSM [1] or ICD [48]) for the diagnosis of SUD (Table 1).

**Table 1:** General description of the included SUD studies (*n* = 21)

Author/ Year	Design	Location	Sample size	Sample age	Sample source, Gender: (M = male, F = female)	SUD diagnostic criteria	Main findings
**Epidemiological description of SUD:**							
Alkhalaf et al., 2019 [46]	Cross sectional (records)	Al-Amal Hospital, Dammam	2048 1993 (*n* = 720), 2013 (*n* = 1328)	Older than 15	Patients (M)	Not mentioned	Significant differences were observed in type of addiction and socio-demographics (age, criminal record, educational level, employment status, relapse, and type of substance abused) between 1993 and 2013.
Ibrahim et al., 2018 [7]	Cross sectional	Psychiatric Rehabilitation Center (PRC), Buraidah	612	(10–80) 73% were 21–40 years	Patients (M)	Not mentioned	Majority were polysubstance users (60%); amphetamine users (24%). No significant association between positive family history of substance abuse or mental and type of substance abused.
Bassiony, 2008 [28]	Cross sectional	Al-Amal hospital in Jeddah	101	(14–61)	Patients (M)	DSM-IV	Adolescents started using drugs at a younger age than adults (*p* = 0.006). Adolescents and adults also differed in the first (*p* = 0.001) and the second (; *p* = 0.002) stages of progression in drug involvement.
AbuMadini et al., 2008 [29]	Cross sectional (records)	Al-Amal Hospital, Dammam	12,743	(83%) were 20–39 years.	Patients (M)	ICD-9 &10	Use increased for amphetamines and cannabis, decreased for heroin, sedatives and volatile substances and remained stable for alcohol.
Abalkhail, 2001 [30]	Cross sectional (Comparative study)	Al-Amal hospital, Jeddah	303	Mean (SD) 30.4 (0.4) heroin, 29.4 (1.0) non-heroin	Patients (M)	Not mentioned	Heroin addicts were seven times more likely to develop hepatitis B or C infection than non-heroin addicts.
Iqbal, 2000 [31]	Cross sectional (records)	Voluntary detoxification unit, Jeddah	799	(17–66) 83% were 20–39 and 68% were under 35.	Patients (M)	DSM-IV	Heroin was the choice of substance (63%) abuse; 14% were poly drug users. Among heroin users, 91% were injecting it and 69% had Hepatitis C.
al-Nahedh, 1999 [32]	Cross sectional	Al-Amal Hospital, Riyadh	160	(20–60) (Mean 29.5)		Not mentioned	Age, unemployment, peer pressure and family and social stresses were significantly associated with repeated admissions.
Hafeiz, 1995 [33]	Cross sectional	Al-Amal Hospital, Dammam.	116	(20–61) 83% were 21–32 year	Patients (M)	DSMIIIR	84% used heroin either alone or in combination with other drugs. 31% used alcohol, 26% used cannabis, and 10% used stimulants. Heroin user were younger and less likely poly-drug abusers than alcoholics.
Osman, 1992 [17]	Cross sectional	Jeddah Psychiatric Hospital	485	(15–65) Mean 29.04 (8.67) 52% were 20–29.	Patients (M, F) (F 2.7%, n = 13)	Substance abuse predefined criteria	43.5% were heroin addicts and 16.1% alcohol abusers; 14.6% were poly-drug abusers. Reasons for drug use: curiosity and peer influence and traveling abroad. 4.9% had criminal history.
Qureshi, 1992 [34]	Cross sectional	Buraidah Mental Hospital	240	–	Patients and controls (M)	DSM III R	Single and/or disruptive marital life, poor social class, unskilled job and/or unemployment and financial issues were significantly associated with drug abusers as contrast to control group (*p* < 0.0001).
Omer and Ezzat, 1999 [47]	Cross sectional (Description of two studies)	Al- Amal hospital, Jeddah	Study A: 43 cases of volatile and similar number control from opiate patients Study B: 50, no comparison control	Older than 15 years	Patients (M)	DSM III R for study A, Not specified for study B	The majority of admitted volatile substance abusers were young, single and unemployed. The two main abused substances were paint and glue. Volatile abusers had no difference in age distribution as compared to the control group of heroin addicts but scored more in the psychosis scale.
Iqbal, 2001 [35]	Cross sectional	Al- Amal hospital, Jeddah	302	> 18 years	adult with substance dependence (M)	DSM-IV	On admission, 57.14% expressed no desire to complete the program. Unaided abstinence was reported by 42.71% and post-treatment abstinence by 57.52%.
**Prevalence and detection of infectious viral diseases among SUD patients:**							
Alshomrani, 2015 [36]	Cross sectional	Alamal Complex for Mental Health in Riyadh, Addiction center.	357	(13–71) Mean (SD) 40 (8.6)	Inpatients heroin users (M)	Not mentioned	Prevalence of HBV surface antigen was 7.7%, antibodies for HCV 77.8%, and HIV 9.8%. A significant association was found between positive HCV and positive HIV tests.
Alzahrani AJ et al., 2009 [37]	Cross sectional	Local rehabilitation center, Dammam	344	–	intravenous drug users (M)	Not mentioned	The prevalence of HBV and HCV was 12% (*n* = 41) and 38% (*n* = 131) compared with prevalence rates in the general population in Saudi Arabia of 1.7 and 5%, respectively. Mixed genomes of HBV, HCV and TTV were observed.
Alzahrani AJ 2008 [38]	Case-Control	Local rehabilitation center, Dammam	Cases (*n* = 297) Control matched blood donors (*n* = 305).	Mean (SD) 31 (2.2)	Patients followed up or admitted to a drug rehabilitation hospital (M)	Not mentioned	The seroprevalence of HBsAg was 6.1%, HCV antibodies was 37.7% (*n* = 112), and HIV antibodies was 0.67% 39% (116) were positive by the new HCV Ag/Ab combination ELISA assay, from which 95% have detectable HCV core Ag.
Alzahrani AJ 2005 [39]	Cross sectional	Local rehabilitation center, Dammam	201	Mean 33	Patients enrolled in drug rehabilitation (M)	Not mentioned	The seroprevalence of HBsAg was 5.9%, the HCV antibodies was 35.6%, and HIV antibodies was 0.99%. Drug users were found to be responsible for approximately 60% of the new cases of HCV infection.
Njoh and Zimmo, 1997 [40]	Cross sectional	Al-Amal Hospital, Jeddah	2628 serum samples	–	Patients admitted for drug dependence (M)	DSM-IV	The overall HIV prevalence of 0.15% (1.5 persons per 1000).
**Factors/ conditions associated with SUD users:**							
Almarhabi et al., 2018 [41]	Cross sectional	Al-Amal hospital addiction center in Jeddah	101	Mean (SD) 33.28 (9.46)	Substance users admitted for rehabilitation (M)	Not mentioned	93% drove under the influence of an abused substance. Current substance use: Amphetamines (38.6%), Cannabis (24.8%). Amphetamines and alcohol were the choice of drug for initiation
Khalawi et al., 2017 [9]	Case control study	Al-Amal Hospital, Jeddah	207 cases 416 controls	Mean (SD) Cases 29.9 (10.9), Controls 33.7 (10.9)	Cases: substance users; (F) Controls: visitors at the primary health center (F)	Not mentioned	Significant risk factors for substance use: presence of family conflicts, substance abuse by husband, substance abuse by peers, substance abuse by siblings, sexual abuse, low family income (*p* < 0.05).
Youssef et al., 2016 [42]	Case control study	Al-Baha Psychiatric Hospital	239 cases, 117 controls	Cases: 18–45 years 31.35 (6.25) Controls 31.66 (7.84)	Cases: patients admitted for substance use; Control: Non drug users (M)	DSM-IV-TR	Amphetamine (87.7%) and cannabis (70.49%) were the most abused substances. Depression and suicide probability are common consequences of substance abuse.
Chinnian et al., 1994 [43]	Case-Control	De-Addiction Hospital, Riyadh	320 experimental (*n* = 250 heroin, *n* = 70 alcohol), 70 medical control 70 normal control	(18–35) Mean: heroin (25.27), alcohol (30.58), control (25.97)	Cases: heroin or alcohol abusers Control 1: patients from general medical trauma wards Control 2: Normal undergraduate students at the Islamic University	Not mentioned	Alcohol abusers scored higher than all the other groups in terms of psychoticism, neuroticism, and anxiety. With the lie scale, the substance-abusing group as a whole recorded significantly higher scores than the controls.
Alzahrani H et al., 2015 [44]	Cross sectional	public health hospital in Jeddah	165	(18–50) 38% were 31–40 years	Inpatients admitted for substance use disorders (M)	Not mentioned	High prevalence of depression (95.2%) among substance users (100% in heroin, 80% in amphetamine users). Prevalence and comorbidity were significantly associated with duration of substance abuse.
**Assessing reliability and validity of a scale among SUD users:**							
Khalil, 2011 [45]	Cross sectional	Al-Amal Hospital of Substance Abuse in Dammam	175	(18–60) Mean (SD) 34.7 (10.2)	Patients from the detoxification and rehabilitation wards (M)	DSM-IV	The Arabic version of the University of Rhode Island Change Assessment (URICA) showed good psychometric properties, supporting the validity and reliability of the four factors of the scale.

Of the total 23 studies, 12 described the demographic characteristics of SUD patients and the types of drugs they used [7, 17, 28–35, 46, 47]; 5 studies were about co-existent conditions of SUD patients, such as anxiety, depression and suicide [9, 41–44]; 5 studies evaluated blood samples of SUD patients in order to determine the prevalence of viral diseases (i.e., HBV, HCV, and HIV) and the accuracy of blood assays [36–40]; 1 study evaluated the psychometric properties of an Arabic version of the University of Rhode Island Change Assessment (URICA) scale with an SUD sample [45].

### Risk (protective) factors of SUD

A total of 4 studies assessed risk factors of SUD. Of them, 3 were cross-sectional and 1 was a case-control study [9, 29, 32, 34]. The case-control study assessed the risk factors among females who use drugs and identified that the SUD patients were more likely to be unemployed, have unstable marriages, less education, low family income, and unstable family conditions than the controls [9]. The cross-sectional studies reported that older age, being single, unemployment, peer pressure, and family and social stresses were associated with SUD [29, 32, 34].

### Substance use and comorbidity

Three studies reported on the relationship between substance use and comorbidity suicide [9, 42–44]. The use of alcohol, amphetamines, and volatile substances showed significant association with suicidal ideation [42]. The use of heroin and amphetamines were significantly associated with depression; all people who used heroin (100%), and 80% of those who used amphetamines reported depression [44]. People who abused alcohol obtained significantly higher scores in the scales of psychosis, neuroticism, and anxiety [43].

### Stages of progression in substance use

Only one study was available on the topic of stages of substance use progression [28]. Adolescents initiated drugs and tobacco use at the same age, while adults, on average, initiated drug use 6 years after tobacco use. In other cultures, alcohol and cannabis are commonly used in the drug initiation phase (gateway hypothesis) [49]. However, the choice of drug in Saudi Arabia is amphetamines for both adolescents and adults.

### Relapse and its predictors

Three studies addressed SUD relapse. In one, older age, unemployment, peer pressure, and family and social stressors were significantly associated with relapse [32]. A second study also found a correlation with the presence of severe psychosocial stressors, but additionally reported the following factors for relapse: heroin dependence, criminal record, divorce, duration of abuse, and family history of addiction [29]; 60% of the patients in this study relapsed within the first 17 months of completion of and discharge from a detoxification/rehabilitation program. The third study found opium and stimulant abuse as significant correlates of relapse [46].

### Perspectives and experiences of drug use

A total of 6 studies described the patterns of drug use among SUD patients [7, 17, 31, 33, 41, 42]; of them, 5 were cross-sectional and 1 was case-control. These studies described the common types of substances being used, number of substances (single versus poly use), and method of administration (inhalation, ingestion, or injection). Curiosity, peer group influence, travelling abroad, and psychiatric disturbances were cited as reasons for the initiation of drug use [17].

### Impact of substance use on family members

No study was found that assessed the impact of substance use on family members of SUD patients. One qualitative study, written in Arabic, was found during the search process. It included in-depth interviews of members from 20 families with a PWUD. They reported rejection of marriage proposal, family isolation from other relatives/friends, financial burden, physical violence, and psychological stress resulting from having a PWUD in the family [50].

### Quality assessment

#### Cross-sectional studies (*n* = 19)

None of the cross-sectional studies were deemed good or very good (0%), 42% were satisfactory, and 58% were unsatisfactory. The areas of weakness identified were non-participation (no study described how many declined to participate and what their characteristics were), sample size (90% of the studies did not provide a sample size calculation), and comparability (84% of the studies did not adjust analyses for confounders). Around 42% did not clearly describe the statistical test that was used including confidence intervals and probability level (*p*-value). The studies scored well in sample representativeness (68% recruited all patients available during the study period) and in the ascertainment of SUD (through the use of diagnostic tools) (Table 2).

**Table 2:** Quality assessment of the included cross-sectional studies using the adopted Newcastle-Ottawa Scale (NOS)

	Selection				Comparability	Outcome		Total
Author	Representativeness of the sample (⋆)	Sample size (⋆)	Non-respondents (⋆)	Ascertainment of the exposure (risk factor) (⋆⋆)	(⋆⋆)	Assessment of outcome (⋆⋆)	Statistical test (⋆)	(*10)
Alkhalaf et al., 2019 [46]	*	–	–	–	–	*	*	3
Ibrahim et al., 2018 [7]	*	–	–	**	**–**	–	–	3
Almarhabi et al., 2018 [41]	*	*	–	–	*	**	*	6
Alshomrani, 2015 [36]	*	–	–	*	**–**	**	*	5
Khalil, 2011 [45]	–	–	–	**	**–**	**	*	5
Alzahrani AJ et al., 2009 [37]	–	–	–	–	**–**	**	–	2
Bassiony, 2008 [28]	–	–	–	**	**–**	*	*	4
Alzahrani AJ, 2005 [39]	*	–	–	–	**–**	**	–	3
AbuMadini et al., 2008 [29]	*	–	–	**	**–**	**	*	6
Abalkhail, 2001 [30]	*	–	–	–	**–**	**	*	4
Iqbal, 2000 [31]	*	–	–	**	**–**	**	–	5
al-Nahedh, 1999 [32]	*	–	–	–	**–**	*	*	3
Njoh and Zimmo, 1997 [40]	*	–	–	**	**–**	**	–	5
Hafeiz, 1995 [33]	*	–	–	**	**–**	*	–	4
Osman, 1992 [17]	*	–	–	*	**–**	**	–	4
Qureshi, 1992 [34]	–	–	–	**	*	**	*	6
Iqbal, 2001 [35]	–	–	–	**	**–**	*	–	3
Alzahrani H et al., 2015 [44]	*	*	–	–	*	**	*	6
Omer and Ezzat, 1999 [47]	–	–	–	*	–	*	*	3

#### Case-control studies (*n* = 4)

Of the 4 case-control studies, 1 was of good, 1 was of satisfactory, and 2 were of unsatisfactory quality. None of the studies (0%) enrolled controls from the community, described the non-participants, or ascertained exposure information from records or blinded interviews. Only 1 study described the criteria it used to define the cases (DSM-IV), and only 2 defined the controls. All studies scored well in the selection of representative cases, similarity of ascertainment, and comparability between cases and controls (Table 3).

**Table 3:** Quality assessment of the included case-control studies using the Newcastle-Ottawa Scale (NOS)

	Selection				Comparability/ confounders	Exposure			Total
Author	Adequacy of case definition (⋆)	Representativeness of the cases (⋆)	Selection of controls (⋆)	Definition of controls (⋆)	Comparability of cases and controls (⋆⋆)	Ascertainment of the exposure (⋆)	Similarity of ascertainment between cases and controls (⋆)	Non-response rate (⋆)	(*9)
Khalawi et al., 2017 [9]	–	*	–	–	**–**	–	*	–	2
Youssef et al., 2016 [42]	*	*	–	*	*	–	*	–	5
Alzahrani AJ, 2008 [38]	–	*	–	–	*	–	*	–	3
Chinnian et al., 1994 [43]	–	*	–	*	*	–	*	–	4

## Discussion

### Summary of results

There have only been a small number of SUD publications in Saudi Arabia until the present. The studies that have been published were weak in quality and deficient in methodology. All the studies were observational, with the majority being cross-sectional. The overwhelming majority of the studies did not include females. Only 1 study was suitable for assessing SUD risk factors, and the few studies that assessed experiences of PWUDs were very limited in their scope of inquiry. No study addressed the impact on family members of having a PWUD in the family.

### Strengths of the included studies

Strengths included robust sample sizes and assessment of comorbid conditions in some studies. All studies enrolled > 100 patients, and a few enrolled > 1000 [29, 40]. Some comorbid conditions that are important in the context of drug use (e.g., viral infections HBV, HCV, and HIV or mental illness, such as anxiety, depression, and suicide) were assessed in several studies [9, 36–44].

### Limitations of the included studies

Most studies on SUD enrolled only patients and did not have a reference group; a few identified peer pressure, substance use by family members, and family problems as the reasons for substance use [4, 46]. However, in the absence of a reference group, these factors cannot be determined with certainty to be the risk factors of SUD. Additionally, all the studies obtained their samples from treatments settings; hence, their findings might not be generalizable. Another limitation was that all but two enrolled male patients only; therefore, the SUD data on Saudi females who use drugs is rather limited. The qualitative study was published in Arabic and is therefore not known to the wider scientific community [50]. In addition, around a quarter of the studies chose disease-oriented outcomes (e.g., co-morbid infection) and not any patient-oriented outcome.

To date, only one review on substance use (Bassiony, 2013) has been published [4]. It included 25 studies, and out of them, approximately 14 were included in the current review. These two reviews reported similar results in terms of types of substance that Saudis use, the comorbid conditions, and factors related to relapse. The current review presented results that were not in the previous review, such as perspectives on drug use by PWUDs, and the impact on family members. Additionally, the quality of the studies was not assessed in the earlier review.

### Knowledge gap

Family and religion are central to Saudi life, yet these have not been assessed in detail in the context of SUD. For example, family size is much larger for typical Saudis than their counterparts in the West, and many Saudi men have multiple wives. How these affect the parent-child relationship is not known. Similarly, parents’ occupation and education, parental and sibling drug use, parental enabling behaviors, and family’s religious environment likely play important roles in the development of substance use among the youngsters.

Data is also missing regarding the relationship between the father and mother when an offspring is using drugs or for that matter between siblings who use drugs and those who do not. Little is also known about the coping strategies that family members adopt to deal with a drug problem in the family or the actions that they take in response, for example, whether they cope differently when the PWUD is the son versus the daughter or how they approach treatment (whether they start rehabilitation immediately or they try alternative methods). Additionally, it is not known whether family members enable the PWUD to continue using drugs, and if so, through which means.

Likewise, very little is known about the experience of PWUDs with drug use. Data is needed on factors related to drug initiation (peer, media, and family influence, age at first use), drug culture (availability, cost, network), knowledge and experience of adverse effects (overdose, signs and symptoms), and treatment-seeking behaviors (cessation attempts and relapses).

Substance use has unwanted consequences, one of which is transmission of diseases through needle sharing or promiscuous sexual behavior. The majority of Saudis who inject drugs are young (< 50 years old), and are therefore able to spread infections to their sexual partners and to other PWUDs with whom they share needles [36]. Consequently, data show that Saudis who inject drugs have a high prevalence of blood borne infections including HIV, HCV, and HBV [36–40, 51]. In order to reduce harm, many western societies have adopted various harm reduction policies, such as needle and syringe exchange programs (NSPs), opioids stimulation therapy (OST), peer distribution of naloxone, overdose response and drug consumption rooms (DCRs), supervised injection facilities, and outreach services for injecting drug users [11, 12]. Saudi Arabia does not have such harm reduction programs in place although it has policies and government-funded programs for treatment and rehabilitation for people with SUD [11].

### Strengths and limitations of this review paper

This review was the outcome of a comprehensive search of multiple databases with specific keywords. Therefore, it is unlikely that it missed any eligible studies. However, it likely missed publications in Arabic and any unpublished data on SUD. In addition, a few screened papers may have been excluded because the sample characteristics of those studies were not clear enough to be assessed for eligibility in this review. Additionally, this review assessed the quality of all included studies using validated scales and identified areas of weakness for these studies.

### Drug use and recent social change

Saudi Arabia has been experiencing rapid social change in recent years. Some of the major changes include the introduction of movie theatres and music concerts, attendance of women at sporting events, permission for women to drive, the replacement of expatriate male workers with Saudi women, and the commencement of tourism. These changes will likely decrease the conservatism and open up the society to outside influences. Saudi youth is already connected, through the internet, to western cultures, which promote drug use and magnify its pleasurable effects. Coupled with this connectivity, these social changes may lead to an unintended increase in substance use.

## Conclusions

The findings of this review underscore the need for rigorous scientific inquiry in multiple domains of SUD. Large epidemiological studies (e.g., case-control) with wider geographical representation are warranted to elicit risk (or protective) factors of SUD, with particular attention given to those that arise from the family environment and/or religious practices. On the other hand, qualitative studies may be more suitable for a better understanding of the universe of SUD patients, including the drug culture in Saudi Arabia or the struggles that family members of a PWUD encounter in their daily lives. Irrespective of study type, there should be an adequate representation of female patients/participants.

The information that can come out of these types of future scientific inquires will be of value when designing community educational and/or awareness campaign so that parents are better informed and better equipped to deal with substance use in the family. Second, this information can be used as the basis for developing and testing interventions aimed at preventing drug initiation, reducing harm, and improving and tailoring rehabilitation programs.

## Supplementary information

**Additional file 1.**

## Data Availability

The datasets used and/or analysed during the current study are available from the corresponding author on reasonable request.

## Abbreviations

CENTRAL: Cochrane Controlled Register of Trials
DCR: Drug consumption room
DSM: Diagnostic and Statistical Manual of Mental Disorders
HBV: Hepatitis B virus
HCV: Hepatitis C virus
HIV: Human immunodeficiency virus
ICD: International statistical classification of diseases and related health problems, 11th revision
NOS: Newcastle-Ottawa Scale
NSP: Needle and syringe exchange program
OST: Opioids stimulation therapy
PWUD: Person who uses drugs
SUD: Substance use disorder
URICA: University of Rhode Island Change Assessment scale

## References

[1] American Psychiatric Association (2013). Substance-Related and Addictive Disorders. Diagnostic and Statistical Manual of Mental Disorders DSM-V. DSM Library.

[2] Butler K, Le Foll B. Impact of substance use disorder pharmacotherapy on executive function: a narrative review. Front Psychiatry. 2019;10(98):1–14. 10.3389/fpsyt.2019.00098 PMC6405638 30881320

[3] United Nations Office on Drugs and Crime (UNODC). World Drug Report 2019. 2019. https://wdr.unodc.org/wdr2019/. Accessed Oct 2019.

[4] Bassiony M (2013). Substance use disorders in Saudi Arabia: review article. J Subst Use.

[5] Alshmrani S. 7% of Saudis are drug users [internet]. Saudi Arabia: Al-Hayat; Newspaper 2017. http://www.alhayat.com/article/812946/. Accessed 15 Sept 2019.

[6] Al-Musa HM, Al-Montashri SDS (2016). Substance abuse among male secondary school students in Abha city, Saudi Arabia: prevalence and associated factors. Biomed Res.

[7] Ibrahim Y, Hussain SM, Alnasser S, Almohandes H, Sarhandi I (2018). Patterns and sociodemographic characteristics of substance abuse in Al Qassim, Saudi Arabia: a retrospective study at a psychiatric rehabilitation center. Ann Saudi Med..

[8] Sweileh WM, Zyoud SH, Al-Jabi SW, Sawalha AF (2014). Substance use disorders in Arab countries: research activity and bibliometric analysis. Subst Abuse Treat Prev Policy.

[9] Khalawi AA, Ibrahim A, Alghamdi AH (2017). Risk factors potentiating substance abuse among Saudi females: a case-control study. Int J Med Res Prof.

[10] World Health Organization (2006). Mental health in the eastern Mediterranean region: reaching the unreached.

[11] World Health Organization (2010). Atlas on substance use (2010): resources for the prevention and treatment of substance use disorders.

[12] Stone K, Shirley-Beavan S (2018). Global state of harm reduction 2018.

[13] Alqarni H. 150 Thousand Addicts in Saudi Arabia with Treatment Expenditure of 3.6 Billion Riyals [Internet]. Saudi Arabia: Al-Riyadh Newspaper; 2013. http://www.alriyadh.com/831719. Accessed 22 Sept 2019.

[14] Staff J, Schulenberg JE, Maslowsky J, Bachman JG, O'Malley PM, Maggs JL (2010). Substance use changes and social role transitions: proximal developmental effects on ongoing trajectories from late adolescence through early adulthood. Dev Psychopathol.

[15] Mellentin AI, Brink M, Andersen L, Erlangsen A, Stenager E, Bjerregaard LB (2016). The risk of offspring developing substance use disorders when exposed to one versus two parent(s) with alcohol use disorder: a nationwide, register-based cohort study. J Psychiatr Res.

[16] LeTendre ML, Reed MB (2017). The effect of adverse childhood experience on clinical diagnosis of a substance use disorder: results of a nationally representative study. Subst Use Misuse.

[17] Osman AA (1992). Substance abuse among patients attending a psychiatric hospital in Jeddah: a descriptive study. Ann Saudi Med.

[18] Alhyas L, Al Ozaibi N, Elarabi H, El-Kashef A, Wanigaratne S, Almarzouqi A (2015). Adolescents' perception of substance use and factors influencing its use: a qualitative study in Abu Dhabi. JRSM Open.

[19] Usher K, Jackson D, O'Brien L (2007). Shattered dreams: parental experiences of adolescent substance abuse. Int J Ment Health Nurs.

[20] Choate PW (2015). Adolescent alcoholism and drug addiction: the experience of parents. Behav Sci (Basel).

[21] General Authority for Statistics Saudi Arabia. Population in Saudi Arabia by age groups and gender - Mid 2019. Statistical yearbook mid 2019. Vol 21. 2019. https://www.stats.gov.sa/en/43. Accessed October 2019.

[22] Njoh J, Zimmo S (1998). Prevalence of antibody to hepatitis D virus among HBsAg-positive drug-dependent patients in Jeddah, Saudi Arabia. East Afr Med J.

[23] Njoh J, Zimmo S (1997). Prevalence of antibodies to hepatitis C virus in drug-dependent patients in Jeddah, Saudi Arabia. East Afr Med J..

[24] Njoh J (1995). Prevalence of hepatitis B virus markers among drug-dependent patients in Jeddah Saudi Arabia. East Afr Med J.

[25] Njoh J (1995). The prevalence of hepatitis B surface antigen (HBsAg) among drug-dependent patients in Jeddah, Saudi Arabia. East Afr Med J..

[26] Herzog R, Alvarez-Pasquin MJ, Diaz C, Del Barrio JL, Estrada JM, Gil A (2013). Are healthcare workers' intentions to vaccinate related to their knowledge, beliefs and attitudes? A systematic review. BMC Public Health.

[27] Takahashi N, Hashizume M (2014). A systematic review of the influence of occupational organophosphate pesticides exposure on neurological impairment. BMJ Open.

[28] Bassiony MM (2008). Stages of progression in drug abuse involvement across generations in Jeddah, Saudi Arabia. Neurosciences (Riyadh, Saudi Arabia).

[29] AbuMadini MS, Rahim SI, Al-Zahrani MA, Al-Johi AO (2008). Two decades of treatment seeking for substance use disorders in Saudi Arabia: trends and patterns in a rehabilitation facility in Dammam. Drug Alcohol Depend.

[30] Abalkhail BA (2001). Social status, health status and therapy response in heroin addicts. East Mediterr Health J.

[31] Iqbal N (2000). Substance dependence. A hospital based survey. Saudi Med J.

[32] al-Nahedh N (1999). Relapse among substance-abuse patients in Riyadh, Saudi Arabia. East Mediterr Health J.

[33] Hafeiz HB (1995). Socio-demographic correlates and pattern of drug abuse in eastern Saudi Arabia. Drug Alcohol Depend.

[34] Qureshi NA (1992). Sociodemographic correlates, pattern and comorbidity of drug abuse among psychiatric patients. Arab J Psychiatr.

[35] Iqbal N (2001). Problems with inpatient drug users in Jeddah. Ann Saudi Med..

[36] Alshomrani AT (2015). Prevalence of human immunodeficiency virus, hepatitis C virus, and hepatitis B virus infection among heroin injectors in the central region of Saudi Arabia. Saudi Med J..

[37] Alzahrani AJ, Dela Cruz DM, Obeid OE, Bukhari HA, Al-Qahtani AA, Al-Ahdal MN (2009). Molecular detection of hepatitis B, hepatitis C, and torque Teno viruses in drug users in Saudi Arabia. J Med Virol.

[38] Alzahrani AJ (2008). Simultaneous detection of hepatitis C virus core antigen and antibodies in Saudi drug users using a novel assay. J Med Virol.

[39] Alzahrani AJ (2005). Analysis of hepatitis C virus core antigenemia in Saudi drug users. Saudi Med J..

[40] Njoh J, Zimmo S (1997). The prevalence of human immunodeficiency virus among drug-dependent patients in Jeddah, Saudi Arabia. J Subst Abuse Treat.

[41] Almarhabi Y, Mufti AI, Almaymuni AD, Abdurahman T, Abdulaziz G, Alghamdi AA (2018). Substance abuse at early age as a potential risk factor for driving under the influence of substance in Jeddah, Saudi Arabia: a cross-sectional study. Traffic Inj Prev.

[42] Youssef IM, Fahmy MT, Haggag WL, Mohamed KA, Baalash AA (2016). Dual diagnosis and suicide probability in poly-drug users. J Coll Physicians Surg Pak.

[43] Chinnian RR, Taylor LR, al Subaie A, Sugumar A, al Jumaih AA (1994). A controlled study of personality patterns in alcohol and heroin abusers in Saudi Arabia. J Psychoactive Drugs.

[44] Alzahrani H, Barton P, Brijnath B (2015). Self-reported depression and its associated factors among male inpatients admitted for substance use disorders in Saudi Arabia. J Subst Use..

[45] Khalil MS (2011). Reliability and confirmatory factor analysis of the arabic version of the University of Rhode Island Change Assessment (URICA). Alcohol Alcohol.

[46] Alkhalaf A, Alahmari T, Ashworth F (2019). Changing trends of substance addiction in Saudi Arabia between 1993 and 2013. MOJ Addict Med Ther.

[47] Omer AA, Ezzat BE (1999). Volatile substance abuse: experience from Al Amal Hospital, Jeddah. Ann Saudi Med.

[48] World Health Organization. International Statistical Classification of Diseases and Related Health Problems, 11th revision (ICD-11). 2018. http://id.who.int/icd/entity/1448597234. Accessed 10 June 2019.

[49] Kandel DB (2002). Stages and pathways of drug involvement: examining the gateway hypothesis.

[50] AlHarbi K (2017). المشكلات التي تواجه أسر مدمني المخدرات في المجتمع السعودي.

[51] Alibrahim OA, Misau YA, Mohammed A, Faruk MB, Ss I (2018). Prevalence of hepatitis C viral infection among injecting drug users in a Saudi Arabian hospital: a point cross sectional survey. J Public Health Afr.

